# Supplementary material for: The interactome of intact mitochondria by cross-linking mass spectrometry provides evidence for coexisting respiratory supercomplexes
Source: Mol Cell Proteomics. 2017 Dec 8;17(2):216–32. doi: 10.1074/mcp.RA117.000470 (PMC5795388; doi:10.1074/mcp.RA117.000470)
Supplement: Supplemental Data [file supp_RA117.000470_133922_0_supp_23978_fzffwf.zip › spectra_annotation/mito_DR_spectra_annotation/427-1-4-1-10-1.pdf]

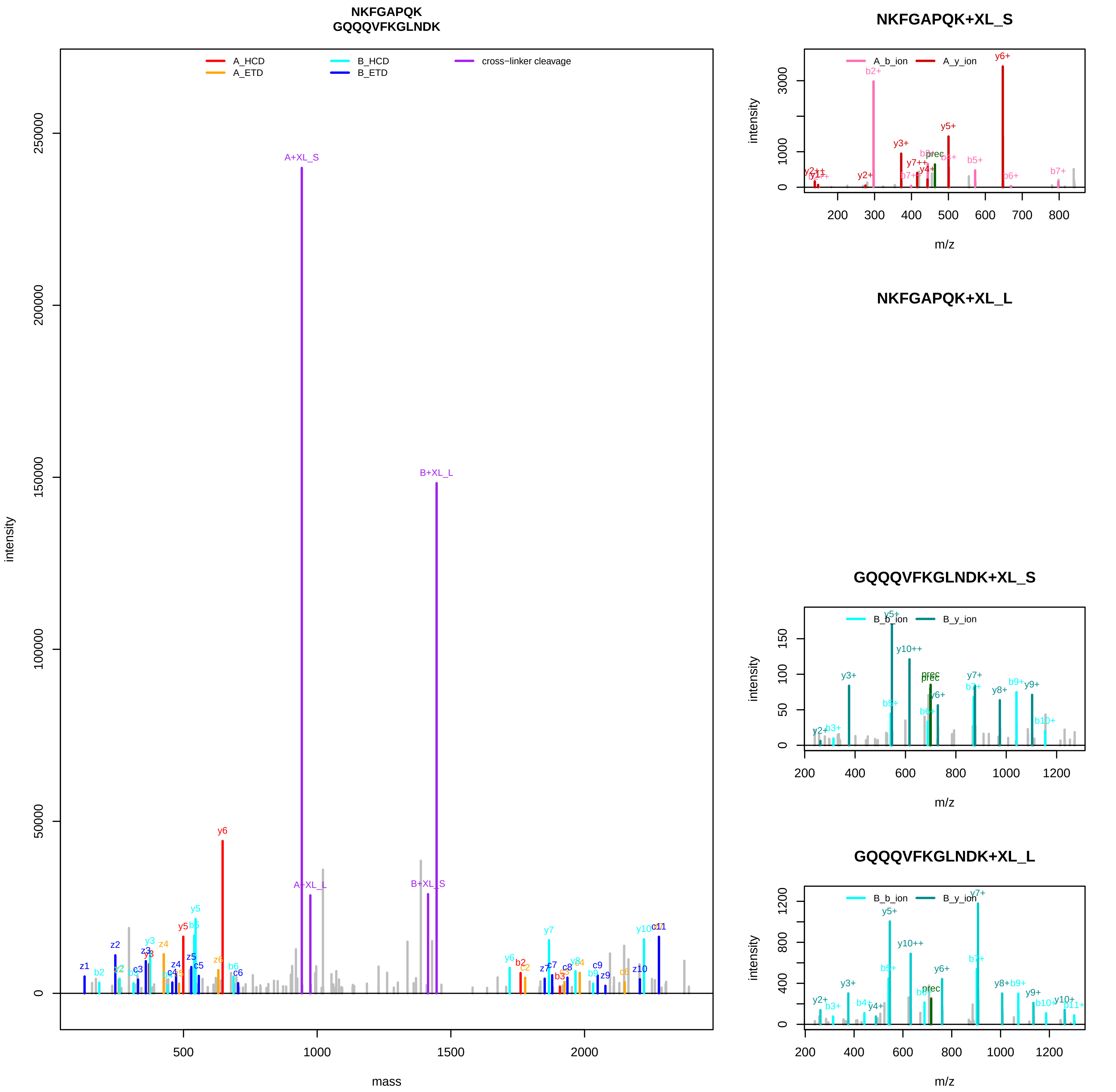
NKFGAPQK
GQQQVFKGLNDK
A_HCD
A_ETD
B_HCD
B_ETD
cross−linker cleavage
A+XL_S
B+XL_L
A+XL_L
B+XL_S
y6
y5
y5
y7
y6
y10
c11
z1
z2
z3
z4
z5
z7
z9
z10
c3
c4
c5
c6
c7
c8
c9
b2
b6
b9
y3
y8
intensity
mass
0
50000
100000
150000
200000
250000
500
1000
1500
2000
NKFGAPQK+XL_S
A_b_ion
A_y_ion
y6+
b2+
y5+
y3+
y2+
b5+
b7+
b6+
prec
intensity
m/z
0
1000
3000
200
300
400
500
600
700
800
NKFGAPQK+XL_L
GQQQVFKGLNDK+XL_S
B_b_ion
B_y_ion
y5+
y10++
y3+
y7+
y8+
b9+
y9+
b10+
y6+
b5+
b6+
b7+
b3+
y2+
prec
intensity
m/z
0
50
100
150
200
400
600
800
1000
1200
GQQQVFKGLNDK+XL_L
B_b_ion
B_y_ion
y7+
y5+
y10++
y6+
b5+
y3+
y8+
b9+
y9+
b10+
y10+
b11+
b4+
y4+
b3+
y2+
b6+
b7+
prec
intensity
m/z
0
400
800
1200
200
400
600
800
1000
1200
